# *Origanum vulgare* ssp. *hirtum* (Lamiaceae) Essential Oil Prevents Behavioral and Oxidative Stress Changes in the Scopolamine Zebrafish Model

**DOI:** 10.3390/molecules26237085

**Published:** 2021-11-23

**Authors:** Luminita Capatina, Edoardo Marco Napoli, Giuseppe Ruberto, Lucian Hritcu

**Affiliations:** 1 Department of Biology, Faculty of Biology, Alexandru Ioan Cuza University of Iasi, 700506 Iasi, Romania; luminita.capatina@student.uaic.ro; 2 Institute of Biomolecular Chemistry, National Research Council ICB-CNR, 95126 Catania, Italy; edoardo.napoli@icb.cnr.it (E.M.N.); giuseppe.ruberto@icb.cnr.it (G.R.)

**Keywords:** *Origanum vulgare* ssp. *hirtum*, essential oil, scopolamine, anxiety, memory, oxidative stress

## Abstract

*Origanum vulgare* ssp. *hirtum* has been used as medicinal herbs promoting antioxidant, anti-inflammatory, antimicrobial, and neuroprotective activities. We investigated the protective effects and the mechanism of *O. vulgare* ssp. *hirtum* essential oil (OEO) on cognitive impairment and brain oxidative stress in a scopolamine (Sco)-induced zebrafish (*Danio rerio*) model of cognitive impairment. Our results show that exposure to Sco (100 µM) leads to anxiety, spatial memory, and response to novelty dysfunctions, whereas the administration of OEO (25, 150, and 300 µL/L, once daily for 13 days) reduced anxiety-like behavior and improved cognitive ability, which was confirmed by behavioral tests, such as the novel tank-diving test (NTT), Y-maze test, and novel object recognition test (NOR) in zebrafish. Additionally, Sco-induced brain oxidative stress and increasing of acetylcholinesterase (AChE) activity were attenuated by the administration of OEO. The gas chromatography–mass spectrometry (GC-MS) analyses were used to elucidate the OEO composition, comprising thymol (38.82%), *p*-cymene (20.28%), and γ-terpinene (19.58%) as the main identified components. These findings suggest the ability of OEO to revert the Sco-induced cognitive deficits by restoring the cholinergic system activity and brain antioxidant status. Thus, OEO could be used as perspective sources of bioactive compounds, displaying valuable biological activities, with potential pharmaceutical applications.

## 1. Introduction

Alzheimer’s disease (AD) is an irreversible and leading cause of 70% of all dementia cases, which include noteworthy, persistent, and progressive memory loss. Additionally, it includes cognitive impairment and personality changes. Drug discovery and development for AD are challenging because no new drug has been approved since 2003 [1]. There are few medications available on the market of acetylcholinesterase inhibitors (AChEIs), such as donepezil (treatment of mild cognitive impairment), rivastigmine, and memantine, which can instigate adverse effects such as vomiting, diarrhea, fatigue, muscle weakness, dizziness, headache, constipation, and so on [2]. In the brain of patients with AD, immoderate reduction in acetylcholine (ACh) hydrolyzed by acetylcholinesterase (AChE) is one of the essential elements in the development of dementia, and an approach in this regard could be through the inactivation of AChE activity, a critical enzyme that cleaves synaptic ACh and stops neuronal signals [3]. 

Zebrafish (*Danio rerio*) is an excellent model for understanding the mechanism of a disease due to its central nervous system, which is organized similarly to vertebrates—traditionally separated into rhombencephalon, mesencephalon, forebrain, ascending and descending spinal cord, cranial nerves, motor spinal cord, and nerves sensory. This animal model has important advantages, including the blood–brain barrier (BBB) of zebrafish, which is structurally and functionally similar and to that of mammals, and many proteins of human associative neurodegenerative diseases have counterparts in zebrafish, highlighting the potential conservatory of molecular cellular functions, which can be easily examined [4]. Locomotion is a complex behavior, as evidenced by zebrafish embryos, which have a basic ability to swim even after hatching. According to behavioral studies, despite the strong association regarding the functions of the brain regions between zebrafish and humans, the neocortex is missing in zebrafish, but the systems of neurotransmitters (dopamine, GABA, glutamate, norepinephrine, serotonin, histamine, and ACh) are present in zebrafish and are responsible for learning and memory and actively participate in the transmission process [5].

The role of cholinergic signaling in acquisition and consolidation has been thoroughly documented [6,7]. Scopolamine (Sco) is a nonselective muscarinic acetylcholine receptor blocker that causes amnesia by impairing learning and short-term memory [8]. Sco causes cognitive impairment in rodents [9] and inhibits memory formation in the object recognition test [10]. Zebrafish is also highly sensitive to Sco, which shows amnesic effects in the Y-maze and novel object recognition (NOR) tests [11,12]. As a result, Sco-induced amnesia models are frequently employed to assess natural products and related substances’ neuroprotective properties.

The Lamiaceae family includes the *Origanum* genus, and most of its species are found in the Mediterranean, Eurasia, and North Africa, where they are used in traditional medicine to treat colds, coughs, stomach, and respiratory disorders [13,14]. It has been documented that *O. syriacum L.* exhibits neuroprotective and beneficial effects in treating several disorders affecting different systems of the body, including the cardiovascular, respiratory, and nervous systems [15]. In addition, it improves learning and memory in AD model mice [16]. The antibacterial, anti-inflammatory, and antioxidant characteristics of *Origanum*’s phenolic components were primarily responsible for its therapeutic impact on traditional medicine [17,18,19]. Several studies have investigated *O. vulgare*’s anti-inflammatory properties in both cell and animal models. Avola et al. [20] demonstrated that *O. vulgare* L. essential oil displays anti-inflammatory activity and facilitates wound healing in a human keratinocytes cell model. Additionally, Han and Parker [21] provided evidence on the anti-inflammatory, tissue remodeling, immunomodulatory, and anticancer activities of *O. vulgare* essential oil in a human skin disease model. Furthermore, Vujicic et al. [22] reported that ethyl acetate extract of *O. vulgare* L. ssp. *hirtum* reduced proinflammatory macrophage/T helper 1/T helper 17 cells response in streptozotocin-induced diabetes in C57BL/6 mice. Antioxidant properties of *O. vulgare* have been extensively investigated, particularly its essential oil derivatives. Kosakowska et al. [23] mainly attributed the antioxidant activity of the essential oils and hydroethanolic extracts of *O. vulgare* L. ssp. *hirtum* Ietswaart and *O. vulgare* L. ssp. *vulgare* to the high amount of carvacrol. Kakhri et al. [24] demonstrated the highest antioxidant activity of the *O. vulgare* essential oil, mainly due to the presence of carvacrol (34.00%) and thymol (35.18%) in its chemical composition.

The chemical composition of the *O. vulgare* essential oil has been extensively studied. Teixeira et al. [25] reported carvacrol (14.50%), thymol (12.60%), *β*-fenchyl alcohol (12.80%), and δ-terpineol (7.50%), following γ -terpinene (11.60%), and α-terpinene (3.70%) as the major compounds detected from the *O. vulgare* essential oil. Methyleugenol (16.50%), myristicin (15.60%), carvacrol (15.00%), thymol (9.80%), and apioline (9.40%) were the major compounds identified by Zhao et al. [26] from *O. vulgare* essential oil. Hamada et al. [27] reported carvacrol (48.38%), thymol (26.55%), γ-terpinene (7.9%), and 1,8-cineol (4.86%) as major identified compounds from *O. vulgare* essential oil. Moreover, carvacrol (71.00%), followed by β-caryophyllene (4.00%), γ-terpinene (4.50%), *p*-cymene (3,50%), and thymol (3.00%) was found to be the major component of *O. vulgare* essential oil [28]. Azizi et al. [29] demonstrated that thymol and carvacrol alleviated cognitive impairments in two rat models of dementia with its anticholinesterase, antioxidant, and anti-inflammatory activities. Additionally, Sudeep et al. [30] demonstrated that β-caryophyllene improved cognitive function in Sco-induced amnesia model mice via the regulation of brain-derived neurotrophic factor and MAPK proteins. To date, no research has shown that *O. vulgare* ssp. *hirtum* protects the memory of zebrafish from Sco-induced cognitive impairment by modulating cholinergic and antioxidant pathways. This study explored the phytochemical composition of the *O. vulgare* ssp. *hirtum* essential oil to see how it affected anxiety, cognitive performance, and brain antioxidant capacity in Sco-induced zebrafish model.

## 2. Results and Discussion

### 2.1. The Chemical Composition of the Origanum vulgare ssp. hirtum Essential Oil

**Figure 1:**
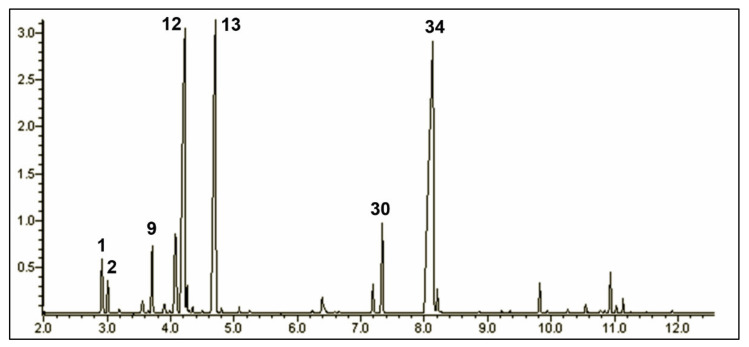
Gas chromatography–mass spectrometry (GC-MS) profile of the *Origanum vulgare* spp. *hyrtum* essential oil (numbers refer to Table 1).

Within the *Origanum* genus, *O. vulgare* is probably more widespread among all species within the genus. Several works confirm a large variability in terms of yields and chemical composition due to a great diversity of factors such as species, soil conditions, harvest season, geographical location, and climatic and growth conditions [31,32]. However, the most common compositional profiles are those that report thymol and/or carvacrol as main components. *O. vulgare* ssp. *hirtum* is largely diffused and important from a commercial perspective. Several studies report a prevalent thymol chemotype for Italian populations [33,34] and a carvacrol chemotype [35]. The chromatographic analyses of the sample used in this study identified 54 compounds covering more than 98% of the total composition. The full composition is reported in Table 1, and the relevant chromatogram is reported in Figure 1. Chemically speaking, the composition of the sample is dominated by monoterpenes (both hydrocarbons and oxygenated), which cover more than 94% of the total composition, followed by sesquiterpenes (3.94%). The main component is thymol (38.82%), and due to the low percentage of carvacrol (0.59%), this essential oil is classified as a thymol chemotype. The other main compounds are *p*-cymene (20.28%) and γ-terpinene (19.58%), the two biosynthetic precursors of thymol. At a much lower percentage, there were two hydrocarbon monoterpenes, α-terpinene (3.51%) and β-myrcene (2.09%). At a non-negligible percentage and a percentage higher than 1%, there were α-thujene (1.52%, monoterpene hydrocarbon), carvacrol methyl ether (3.11%, oxygenated monoterpene), and β-bisabolene (1.27%, sesquiterpene). The results are in accordance with Pasias et al. [36], who revealed two main compounds, carvacrol (74.20%) and *p*-cymene (8.20%), in the case of *O. vulgare* essential oil analysis. Qiao et al. [37] reported that the main constituents of *O. vulgare* essential oil are phenols carvacrol (75.72%) and thymol (2.44%). Additionally, Zhao et al. [38] showed methyleugenol (16.50%), myristicin (15.60%), carvacrol (15.00%), thymol (9.80%), apioline (9.40%), and (*Z*)-β-farnesene (8.7%), as the major component from *O. vulgare* essential oil. Furthermore, Kosakowska et al. [23] reported the presence of carvacrol (37.21%) as the most abundant, followed by γ-terpinene (17.21%) and *p*-cymene (11.13%), in the chemical composition of *O. vulgare* ssp. *hirtum* essential oil. Based on these results, our essential oil shows a chemical composition comparable to those mentioned by other authors who assume its memory-enhancing and antioxidant function.

### 2.2. Improvement of Anxiety-Like Behavior, Spatial Memory, and Response to Novelty in the NTT, Y-Maze and NOR Tests

Novelty-based paradigms are commonly used in behavioral neuroscience to study affective (fear, anxiety) and cognitive (habituation) phenomena. Fish behavior was assumed to be instinctively driven, with little cognitive ability, but after the many studies that have been conducted on this animal model so far, it was concluded that zebrafish are capable of forming spatial memories and cognitive maps. The NTT test uses vertical distribution in a novel environment as a validated behavioral test for assessing anxiety-like behavior in adult zebrafish. This test uses zebrafish’s instinctive behavior of seeking protection in novel environments, which is conceptually similar to the rodent open field [39]. Additionally, studies showed that the NTT test exploited the natural tendency of zebrafish, diving initially to the bottom of the new experimental tank, with a gradual increase in vertical activity over time [40].

**Figure 2:**
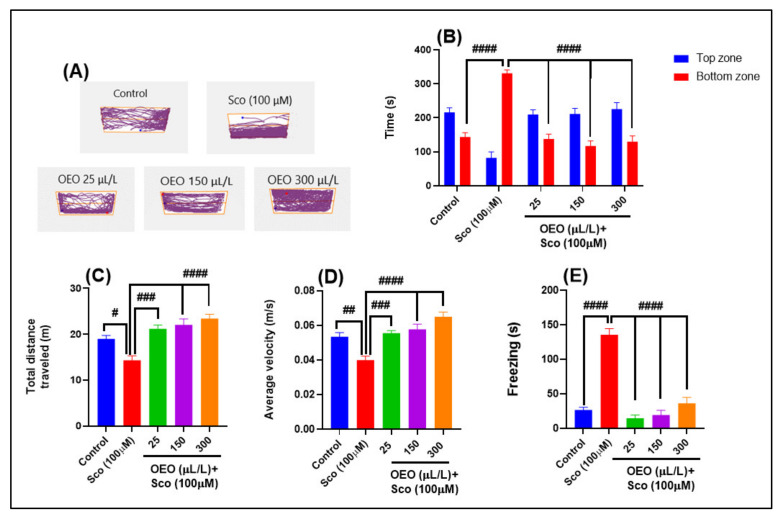
*Origanum vulgare* ssp. *hirtum* essential oil (OEO: 25, 150, and 300 μL/L) improved locomotion pattern and reduced anxiety in the NTT test: (**A**). Locomotion tracking patterns of the control, scopolamine (Sco: 100 μM), and OEO (25, 150, and 300 μL/L) treated groups; (**B**). The time spent in the top/bottom zone by zebrafish in the tank in different groups; (**C**). The total distance traveled (m) by zebrafish in different groups; (**D**). The average velocity (m/s) of zebrafish in the tank in different groups; (**E**). The freezing duration (s) of zebrafish in the tank in different groups. Values are means ± S.E.M. (*n* = 10). For Tukey’s post hoc analyses: # *p* < 0.01, ## *p* < 0.001, ### *p* < 0.0001, and #### *p* < 0.00001.

Representative locomotion-tracking patterns (Figure 2A) in Sco-induced zebrafish indicated a high level of anxiety, as evidenced by their increased exploration of the bottom zone of the tank compared to the control group. Moreover, the improved exploration in the OEO-treated groups pretreated with Sco was noticed, as opposed to the Sco-treated group.

One-way ANOVA indicated significant overall changes in the time spent in the top/bottom zone [F (4, 90) = 48.96, *p* < 0.0001] (Figure 2B). Figure 2B shows the differences in exploring the two zones of the novel tank, with a significant decrease in the exploration time of the bottom zone in the OEO-treated groups over the Sco-treated group (*p* < 0.0001). The Sco-treated group explored the bottom zone of the tank several times, which indicated the anxiogenic profile, as evidenced by a significant increase in the exploration time of the bottom zone compared to the control group (*p* < 0.0001). The other two parameters representative for the NTT test that reflect the locomotor activity of zebrafish are the total distance traveled by zebrafish within the novel tank and average velocity that is magnitude and direction of zebrafish speed. An increase or decrease in velocity reflects the motor aspects of zebrafish swimming, while the total distance travel reflects the general motor and neurological phenotypes [41].

One-way ANOVA indicated significant overall changes in the total distance traveled [F (4, 45) = 13.22, *p* < 0.0001] (Figure 2C) and the average velocity [F (4, 45) = 14.04, *p* < 0.0001] (Figure 2D). Sco treatment induced a hypolocomotor effect by decreasing the total distance traveled (Figure 2C) (*p* < 0.01) and the average velocity (Figure 2D) (*p* < 0.001) compared to the control group. Moreover, OEO treatment dose-dependently prevented Sco-induced hypolocomotion (*p* < 0.0001 for 25 μL/L and *p* < 0.00001 for 150, and 300 μL/L) compared to the Sco-alone treated zebrafish, implying that it has anxiolytic properties.

Freezing duration is another NTT parameter (Figure 2E), which describes the total duration of all freezing bouts. One-way ANOVA indicated significant overall changes in the freezing duration [F (4, 45) = 53.97, *p* < 0.0001] (Figure 2E). For the Sco-treated group, the freezing duration had a high activity compared to the control group (*p* < 0.00001) (Figure 2E), which indicates immobility and anxiety, while OEO promoted a significant reduction in the freezing sessions, suggesting anxiolytic properties.

Our results agree with the literature data where the administration of the *O. vulgare* essential oil reduced depressive-like behavior. Amiresmaeili et al. [42] reported that *O. vulgare* essential oil alleviated depressive symptoms in a rat model of chronic unpredictable stress. Abbasi-Maleki et al. [43] demonstrated that *O. majorana* essential oil shows antidepressant-like effects through involvement with dopaminergic (D_1_ and D_2_), serotonergic (5HT1_A_, 5-HT2_A_ receptors) and noradrenergic (α_1_ and α_2_ adrenoceptors) systems. Rezaie et al. [44] demonstrated the anxiolytic effects of the *O. majorana* extract with diazepam in rats, which was mainly attributed to the interaction between the flavonoids from the extract and GABA-A receptors. Mombeini et al. [45] suggested that the aqueous extract of the *O. vulgare* leaves and flowers proved anxiolytic-like and sedative effects in rats with no myorelaxant effect. Machado et al. [46] demonstrated that β-caryophyllene from the *O. vulgare* exhibited anxiolytic effects in the elevated plus maze test when administered to Swiss mice.

GABAergic neuro-inhibition is known to be potentiated by anxiolytic medications. Flavonoids are phytoconstituents that affect GABA_A_ receptors, the brain’s most important inhibitory receptors, and hence exert an anxiolytic-like effect by inhibiting neuronal activity via GABA [47]. The obtained results demonstrate that OEO exhibits a remarkable anxiolytic behavior. The positively anxiolytic effects of the OEO depend on the activity of its identified chemical constituents, which might explain the mechanism of action. Bianchini et al. [48] reported that thymol exhibited GABAergic activity through interaction with GABA_A_ receptors. Dougnon and Ito [49] demonstrated that the GABAergic system mediated the sedative activity of *p*-cymene through interaction with GABA_A_ receptors. Additionally, Wang and Heinbockel [50] demonstrated that γ-terpinene exhibits anxiolytic-like activity by targeting the GABAergic system.

On these findings, our results indicate that *O. vulgare* ssp. *hirtum* essential oil could reverse the Sco-induced anxiety in the zebrafish model by modulating of the GABAergic system activity.

The Y-maze test has been used with great success in rodents for assessing learning and memory functions [51]. Therefore, for zebrafish, the Y-maze test also assists in the assessment of learning and memory functions and evaluates the effects of pharmacological interventions [11].

**Figure 3:**
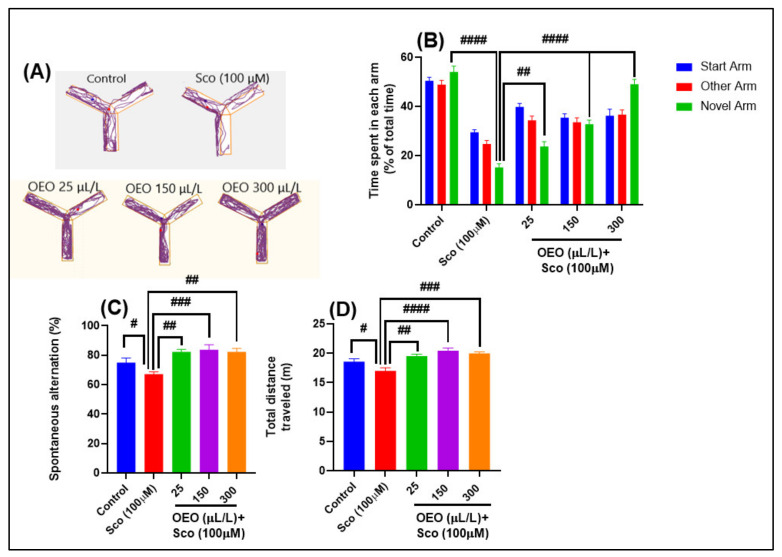
*Origanum vulgare* ssp. *hirtum* essential oil (OEO: 25, 150, and 300 μL/L) improved spatial memory and exploratory behavior in the Y-maze test: (**A**). Locomotion tracking patterns of the control, scopolamine (Sco: 100 μM), and OEO (25, 150, and 300 μL/L) treated groups. (**B**). Time spent in each arm (% of the total time) by zebrafish in the tank in different groups; (**C**). Spontaneous alternation (%) in different groups; (**D**). The total distance traveled (m) by zebrafish in the tank in different groups. Values are means ± S.E.M. (*n* = 10). For Tukey’s post hoc analyses: # *p* < 0.01, ## *p* < 0.001, ### *p* < 0.0001, and #### *p* < 0.00001.

Representative tracking plots of the zebrafish exposed to Sco indicated deficits in exploring novel arm of the Y-maze (Figure 3A). Moreover, an increase in the exploration of the novel arm following administration of OEO was noticed. One-way ANOVA indicated significant overall changes in the time in the novel arm [F (8, 135) = 13.00, *p* < 0.0001] (Figure 3B). OEO (25, 150, and 300 μL/L) benefits are represented by the time spent in each arm (start, other, and novel arm) in different groups, in which the most major interest is in the good exploration time of the novel arm (*p* < 0.001 for 25 μL/L and *p* < 0.00001 for 150 and 300 μL/L), as compared to Sco-alone treated zebrafish. The reduced percentage of the time spent in the novel arm suggests a memory impairment effect in the Sco-induced zebrafish (*p* < 0.00001) compared to the control group.

Spontaneous alternation behavior (Figure 3C) describes the tendency of animals to alternate their turn direction in consecutive turns, and unlike other amnestic tasks, this does not require any prior training or reinforcement. One-way ANOVA revealed significant overall changes in the percentage of spontaneous alternation [F (4, 45) = 7.32, *p* < 0.0001] (Figure 3C). Sco administration caused a significant reduction in spatial memory, as evidenced by significant reduction in the spontaneous alternation percentage (*p* < 0.01) compared to the control group. Additionally, the administration of OEO (25, 150, and 300 μL/L) in pretreated Sco zebrafish improved cognitive status by increasing the spontaneous alternation percentage compared to Sco zebrafish treated only with Sco (*p* < 0.001 for 25 and 300 μL/L and *p* < 0.0001 for 150 μL/L).

One-way ANOVA indicated significant overall changes in locomotion (total distance traveled) [F (4, 45) = 10.22, *p* < 0.0001] (Figure 3D). Sco treatment affects locomotion, a fact proven for total distance traveled (*p* < 0.01), as compared to the control group. Sco-treated zebrafish exposed to OEO (25, 150, and 300 μL/L) exhibited improvement in locomotor activity, as evidenced by a significant increase in the total distance traveled (*p* < 0.001 for 25 μL/L, *p* < 0.00001 for 150 μL/L and *p* < 0.0001 for 300 μL/L).

**Figure 4:**
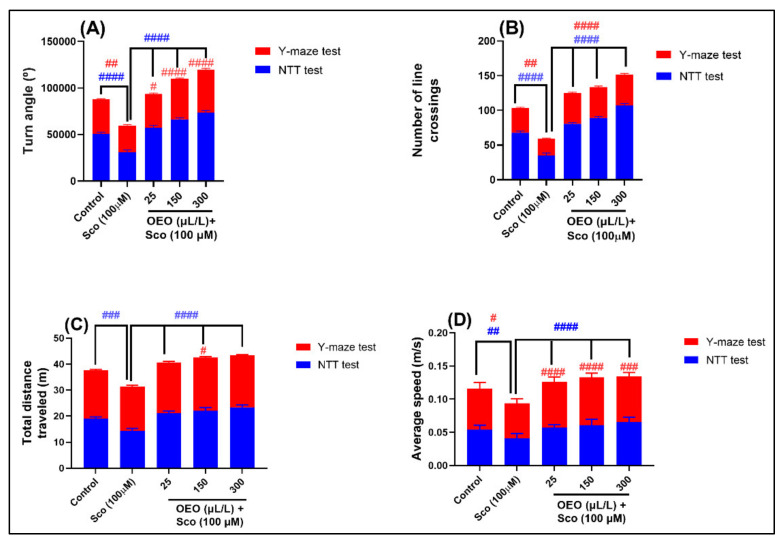
Comparative analyses of zebrafish anxiety-like behavior and spatial memory for the control, Sco (100 μM) and OEO (25, 150, 300 µL/L) group treatment, based on locomotion parameters for the novel tank diving test (NTT) vs. the Y-maze test. (**A**). Turn angle (°); (**B**). Number of line crossings; (**C**). Total distance traveled (m); (**D**). Average speed (m/s). Values are means ± S.E.M. (*n* = 10). For Tukey’s post hoc analyses: # *p* < 0.01, ## *p* < 0.001, ### *p* < 0.0001, and #### *p* < 0.00001.

It has been shown that zebrafish have a remarkable capacity to perform learning tasks. We used NTT to represent the most used zebrafish anxiety models, which focuses on zebrafish diving in response to potentially threatening stimuli, whereas the Y-maze is based on zebrafish spatial memory, as determined by memorized geometric indicators. In our study, we aimed to demonstrate a good cross-test correlation in vivo with the NTT and Y-maze behavior, both tests having similar sensitivity to locomotory zebrafish’s anxiety-like status. Both the NTT and Y-maze, while although different in the workloads set out in each protocol, still characterize the motor capacity of zebrafish. We selected the common parameters to compare the average of the values obtained in the two tests on a common graph. According to Figure 4, NTT evoked high levels for all locomotor parameters, such as the turn angle (*p* < 0.00001) (Figure 4A), number of line crossings (*p* < 0.00001) (Figure 4B), total distance traveled (*p* < 0.00001) (Figure 4C), and average speed (*p* < 0.00001) (Figure 4D), comparative with the Y-maze, which evoked significant higher levels for the turn angle (*p* < 0.00001) (Figure 4A), number of line crossings (*p* < 0.00001) (Figure 4B), and average speed (*p* < 0.00001) (Figure 4D) in the OEO-exposed groups pretreated with Sco. Finally, we can affirm that NTT and Y-maze tests affect the locomotory anxiety-like status in zebrafish and emphasize their developing utility and importance for neurobehavioral research.

**Figure 5:**
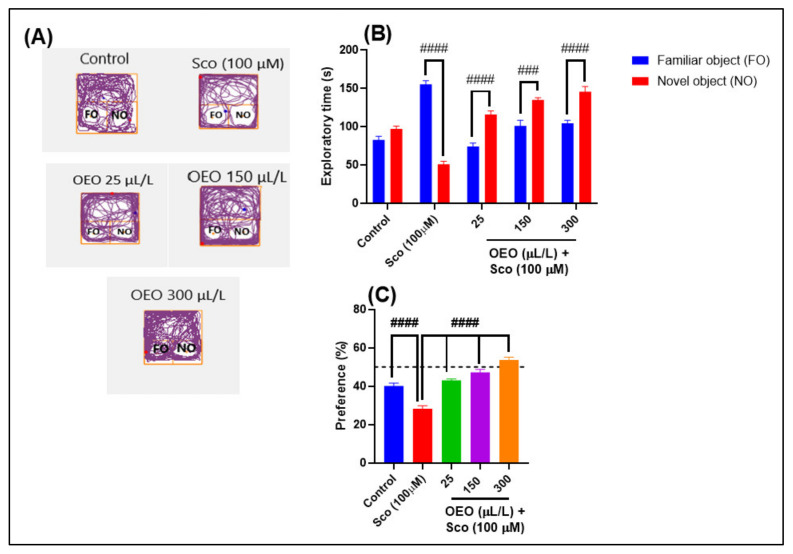
*Origanum vulgare* ssp. *hirtum* essential oil (OEO: 25, 150, and 300 μL/L)-improved memory in the novel object recognition (NOR) test. (**A**): Locomotion tracking patterns of the control, scopolamine (Sco: 100 μM), and OEO (25, 150, and 300 μL/L)-treated groups; (**B**). The exploratory time (s) in different groups; (**C**). The percentages of preference in different groups. Values are means ± S.E.M. (*n* = 10). For Tukey’s post hoc analyses: ### *p* < 0.0001 and #### *p* < 0.00001.

Previous studies demonstrated that the telencephalon (with subdivisions homologous to the hippocampus and mammalian amygdala) is the area responsible for learning and memory in teleost fish. The NOR test is useful for studying both short-term and long-term memory. In this test, avoidance learning is inferred by the amount of time spent outside the compartment previously associated with an aversive stimulus. Passive avoidance learning is frequently used to characterize associative learning and short- and long-term memory in zebrafish used to describe the effects of Sco. By simply manipulating the retention interval, which is the amount of time between training and test sessions, it is possible to evaluate either type of memory [52]. Representative locomotion-tracking patterns illustrated the differences between exploring a familiar object (FO) and the novel object (NO) for each group in the NOR test (Figure 5). As can be seen in Figure 5A, the great preference for FO was found mainly in the Sco-induced zebrafish group, whereas the control group and the groups exposed to OEO have a great preference in exploring the NO.

For the NOR test, one-way ANOVA revealed significant overall effects on exploratory time [F (4, 90) = 84.07, *p* < 0.0001] (Figure 5B) and preference percentage (F (4,45) = 46.14, *p* < 0.0001) (Figure 5C). Regarding the exploratory time (Figure 5B), Sco-treated zebrafish exhibited a high preference to explore FO compared to NO (*p* < 0.0001), thus suggesting deficits of the recognition memory. The Sco-induced zebrafish exposed to 25 and 300 μL/L OEO explored for more time (*p* < 0.00001) NO than FO, suggesting a cognitive-enhancing profile. According to Figure 5C, the lowest percentage for the preference of NO is obviously found in the group treated with Sco (*p* < 0.00001), while an excellent percentage for the NO preference in the OEO (25, 150, 300 µL/L) group treatment was identified.

**Figure 6:**
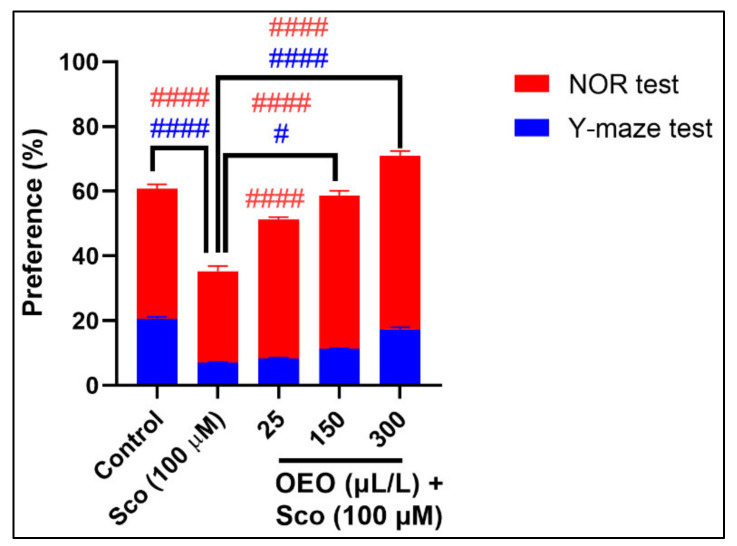
Comparative analyses of zebrafish preference for novelty behavior for the control, Sco (100 μM) and OEO (25, 150, 300 µL/L) group treatment, based on spatial memory parameters for the novel object recognition test (NOR) vs. the Y-maze test. Values are means ± S.E.M. (*n* = 10). For Tukey’s post hoc analyses: # *p* < 0.01 and #### *p* < 0.00001.

For the Y-maze and NOR tests, spatial memory and response to novelty were evaluated, either by exploring the novel arm or exploring the novel object. In our study, we aimed to demonstrate a good cross-test correlation in vivo, between those mentioned test’s behavior, both tests having similar sensitivity to novelty response zebrafish memory-like status (Figure 6).

According to Figure 6, the NOR test evoked higher levels for the preference of the novel object than the control group and OEO (25, 150, and 300 μL/L) -treated groups, as compared to the Sco-treated group (*p* < 0.00001). Moreover, the Y-maze test showed significant differences in the case of the control group and groups exposed to OEO (300 μL/L) (*p* < 0.00001) compared to the Sco-induced group. We can conclude that the Y-maze and NOR tests influenced the spatial memory of zebrafish by responding to novelty through both geometric shapes (square, tringles, and circles) and cubes, emphasizing the usefulness and importance of these behavioral tests in neurobehavioral research on laboratory animals.

Our findings show that *Origanum vulgare* ssp. *hirtum* essential oil has a cognitive-enhancing profile, which is consistent with previous research showing that this essential oil greatly reduces memory deterioration. Ghaderi et al. [53] demonstrated that the aqueous extract of *O. vulgare* enhanced learning and memory in rats. Haghpanah et al. [54] demonstrated that the intra-hippocampal injection of *Origanum* aqueous extract improved rat working memory. Maryam et al. [55] showed that that extract of *O. vulgare* with antioxidant effect-improved working and reference memory impairment. Sheibani et al. [56] demonstrated that the effect of the aqueous extract of *O. vulgare* L. ssp. *viridis* improved the discrimination learning and LTP induction in the CA1 region of the rat hippocampus. These findings show that *Origanum* oil, which was studied in this study, can increase exploratory behavior and recognition memory function in the Sco zebrafish model, as demonstrated in the current study. Additionally, OEO sustained the improvement of spatial memory due to its potent cognitive-enhancing activities of the major compounds (thymol, *p*-cymene and γ-terpinene). Asadbegi et al. [16] demonstrated that thymol attenuated learning and memory impairment induced by intrahippocampal injection of amyloid beta peptide in high fat diet-fed rats. Additionally, Seifi-Nahavandi et al. [57] reported that *p*-cymene improved memory performance in an Aβ1-42-iduced a rat model of AD. Furthermore, Kim et al. [58] demonstrated a memory-enhancing effect of the γ-terpinene in amnesic mice, one of the identified compounds from the *Abies koreana* essential oil. Our research establishes a solid basis for the use of the OEO in the amelioration of memory loss and dementia.

### 2.3. In Vivo Inhibitory Activity against Acetylcholinesterase Activity

The levels of biochemical parameters linked to cholinergic functions, such as acetylcholinesterase (AChE), were examined to clarify the underlying mechanism of OEO’s memory enhancement behavior in Sco-treated zebrafish.

**Figure 7:**
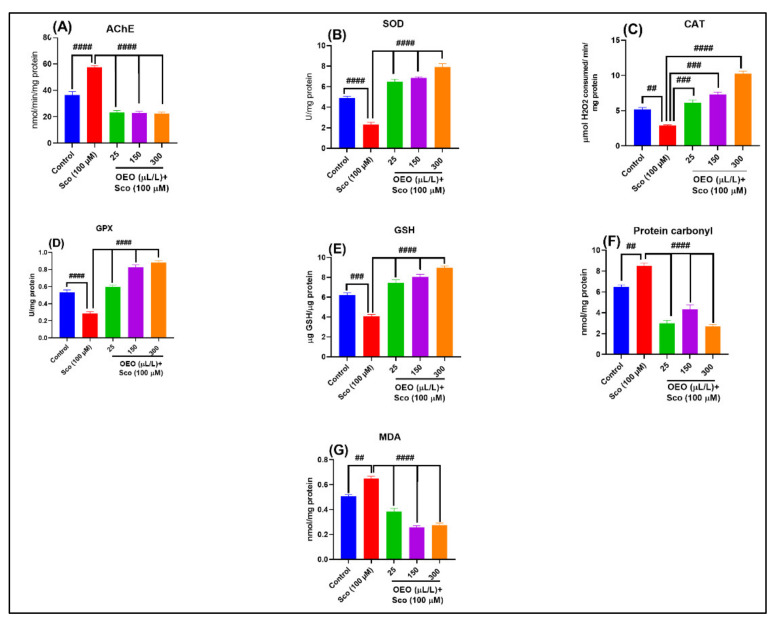
Antioxidant effects of *Origanum vulgare* ssp. *hirtum* essential oil (OEO: 25, 150, and 300 μL/L) in scopolamine (Sco, 100 μM)-induced memory impairment zebrafish brains: (**A**). acetylcholinesterase (AChE); (**B**). Superoxide dismutase (SOD); (**C**). Catalase (CAT); (**D**). Glutathione peroxidase (GPX); (**E**). Reduced glutathione; (**F**). Protein carbonyl; (**G**). Malondialdehyde (MDA). Values are means ± S.E.M. (*n* = 10). For Tukey’s post hoc analyses: ## *p* < 0.001, ### *p* < 0.0001, and #### *p* < 0.00001.

The results of the on-way ANOVA demonstrated overall significant effects [F (4, 45) = 94.86, *p* < 0.0001] on the AChE activity (Figure 7A). AChE activity, which inhibits the synthesis of acetylcholine (ACh), was highest in the Sco-treated group (*p* < 0.00001) compared to control group. Interestingly, AChE activity was significant in all OEO (25, 150, 300 µL/L) groups (*p* < 0.00001) compared to the Sco-alone treated group. This means that the administration of OEO effectively inhibited AChE activity.

Supporting evidence demonstrated the AChE inhibitory activity of the *O. vulgare*. Important AChE inhibitory activity of *O. vulgare* L. grown in Supra Mediterranean region (IC_50_ = 73.7 ± 0.5 μL/L) and Meso-Mediterranean region (IC_50_ = 61.5 ± 0.5 μL/L) was observed [59,60]. Sarikurcku et al. [61] reported the AChE inhibitory effect of essential oils derived from two species of *O. vulgare* L.: *O. vulgare* subsp. *vulgare* and *O. vulgare* subsp. *hirtum*, harvested from Turkey. The authors attributed these effects to the high amounts of thymol, carvacrol, and linalool. According to these findings, our OEO improved memory processes in Sco-induced zebrafish by restoring cholinergic function, meaning that AChE activity was inhibited. Thus, OEO decreased the cholinergic deficits generated following Sco administration, which, as a result, enhanced nootropic action in Y-maze and NOR tests.

### 2.4. In Vivo Antioxidant Activity

Furthermore, we assessed the effects of OEO on antioxidant factors such as SOD, CAT, and GPX-specific activities, reduced GSH levels and contents of protein carbonyl and MDA in Sco-induced memor- impaired zebrafish brain tissue. The results of the one-way ANOVA revealed overall significant differences in SOD [F (4, 45) = 96.61, *p* < 0.0001] (Figure 7B), CAT [F (4, 45) = 78.61, *p* < 0.0001] (Figure 7C), and GPX [F (4, 45) = 93.36, *p* < 0.0001] (Figure 7D) -specific activities and reduced GSH levels [F (4, 45) = 65.25, *p* < 0.0001] (Figure 7E). Sco treatment resulted in a significant decrease in the specific activities of antioxidant enzymes, possibly further increasing the oxidative damage of SOD (*p* < 0.00001) (Figure 7B), CAT (*p* < 0.001) (Figure 7C), and GPX (*p* < 0.00001) (Figure 7D), and the reduced content of GSH (*p* < 0.0001) (Figure 7E) as compared to the control group. The OEO groups had significantly enhanced antioxidant SOD (*p* < 0.00001) (Figure 7B), CAT (*p* < 0.0001 for 25 and 150 μL/L, and *p* < 0.00001 for 300 μL/L) (Figure 7C), GPX (*p* < 0.00001) (Figure 7D) -specific activities and reduced GSH levels (*p* < 0.00001) (Figure 7E) compared to the Sco-alone treated zebrafish. Furthermore, one-way ANOVA revealed significant overall differences in protein carbonyl [F (4, 45) = 70.65, *p* < 0.0001] (Figure 7F) and MDA [F (4, 45) = 84.59, *p* < 0.0001] (Figure 7G) levels. Levels of protein carbonyl (protein carbonyl) (Figure 7F) and MDA (lipid peroxidation) (Figure 7G) were significantly increased (*p* < 0.001) in Sco-treated zebrafish compared to the control groups. Additionally, the Sco-treated zebrafish co-administered with OEO had significantly lower protein carbonyl (*p* < 0.0001) (Figure 7F) and MDA (*p* < 0.0001) (Figure 7G) compared to the zebrafish treated with Sco alone, confirming the antioxidant effects of OEO.

The etiology of AD is complicated by oxidative stress [62]. In Sco-induced memory impairment mice and human patients with AD, levels of SOD and GSH in the antioxidant defense system are significantly reduced [63,64]. The literature data supported that *Origanum* exhibited antioxidant profile. Sharifi-Rigi et al. [65] reported that it had inhibitory effects on paraquat-induced liver damage due to its antioxidant properties. Additionally, Zou et al. [66] demonstrated that oregano essential oil exhibited protection against H_2_O_2_-induced IPEC-J2 cell damage by inducing Nrf2 and related antioxidant enzymes. Sun et al. [67] demonstrated that *O. vulgare* extract ameliorated finasteride-induced hepatic and renal biochemical and histopathological alterations in mouse liver and kidney and restored the antioxidant/oxidant balance. Furthermore, the antioxidant activity of our OEO could be attributed to the high presence of thymol (38.82%), *p*-cymene (20.28%), and γ-terpinene (19.58%), each one having the property to form chemical complexes with metal ions and free radicals [68]. Thymol exhibits a higher antioxidant activity, as reported by Siddiqui et al. [69]. Additionally, the antioxidant effects of *p*-cymene were reported by Formiga [70]. Moreover, the antioxidant capacity of γ-terpinene was presented by Memari-Tabrizi et al. [71]. The present data demonstrate that OEO showed antioxidant properties due to phenolic constituents and thus could be an alternative supply of natural antioxidants for therapeutic purposes.

### 2.5. Correlation between Behavioral Scores, Enzymatic Activities, and Lipid Peroxidation

**Figure 8:**
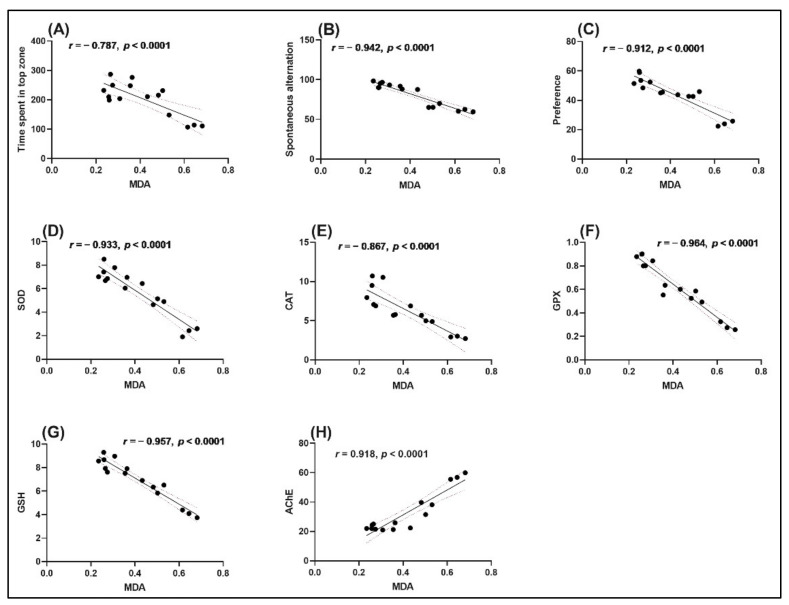
Correlation analyses between behavioral and biochemical parameters (Pearson’s correlation). (**A**). Time spent in top zone vs. MDA; (**B**). Spontaneous alternation vs. MDA; (**C**). Preference vs. MDA; (**D**). SOD vs. MDA; (**E**). CAT vs. MDA; (**F**). GPX vs. MDA; (**G**). GSH vs. MDA; (**H**). AChE vs. MDA. Data expressed are time in tope zone (s), spontaneous alternation %, preference %, SOD (U/mg protein), CAT (μmol H_2_O_2_ consumed/min/mg protein), GPX (U/mg protein), GSH (μg GSH/μg protein), AChE (nmol/min/mg protein), and MDA (nmol/mg protein).

The relation between behavioral scores, enzymatic activity, and lipid peroxidation was evaluated using Pearson’s correlation coefficient (*r*), including the time spent in the top zone, spontaneous alternation, preference, SOD, CAT, GPX, GSH, and AChE (Figure 8). The time spent in top zone (Figure 8A), spontaneous alternation (Figure 8B), preference (Figure 8C), SOD (Figure 8D), CAT (Figure 8E), GPX (Figure 8F), and GSH (Figure 8G) showed a significant negative correlation with MDA with *r* of −0.787 (Figure 8A), −0.942 (Figure 8B), −0.912 (Figure 8C), −0.933 (Figure 8D), −0.867 (Figure 8E), −0.964 (Figure 8F), and −0.957 (Figure 8G), respectively. Additionally, a strong positive correlation between AChE vs. MDA (Figure 8H) was identified with *r* of 0.918 (Figure 8H).

Morshedloo et al. [72] demonstrated a strong correlation between the chemical composition and antioxidant activity of essential oils in *O. vulgare* ssp. *gracile*. Additionally, Qneibi et al. [73] reported a positive correlation between the chemical composition of *O. syriacum* L. essential oil and its neuroprotective potential through its effects on AMPA receptors. The data (*r* values) were used to show that increased memory output in Sco-treated zebrafish is linked to increased antioxidant enzyme activity and decreased MDA (lipid peroxidation levels), validating the neuroprotective profile of OEO.

## 3. Materials and Methods

### 3.1. Essential Oil and Chemical Material

The *O. vulgare* ssp. *hirtum* essential oil used in this study was a commercial sample produced with organic plant material and kindly supplied by Flora S.R.L. (Lorenzana, Pisa, Italy), batch no. 171025. Standard mix of *n*-alkanes C_9_-C_22_ was purchased from Alltech (Italy).

### 3.2. Gas Chromatograph–Mass Spectrometry (GC-MS) Analysis

Gas chromatographic (GC) analysis of the *O. vulgare* ssp. *hirtum* essential oil was conducted using a GC-17A gas chromatograph (Shimadzu, Milan, Italy) equipped with a fused silica ca-pillary column (Supelco SPBTM-5 15m, 0.1mm, 0.1mm, Merck KGaA, Darmstadt, Germany) and Flame Ionization Detector (FID) as the detector. GC–MS analyses were performed on GCMS-QP5050A (Shimadzu, Milan, Italy) The operating conditions for both runs were the following: 60 °C for 1 min, 60–280 °C at 10 °C/min then 280 °C for 1 min; injector temperature 250 °C; detector temperature 280 °C; carrier gas helium (1 mL/min); volume of injection 1 μL (4% essential oil/CH_2_Cl_2_ *v/v*). Percentages of compounds were determined from their peak areas in the GC-FID profiles. Mass spectrometer parameters were the following: ionization at 70 eV, Ion source temperature of 180 °C. Mass spectral data were acquired in the scan mode in *m/z* range 40–400. Oil solutions were injected into the split mode (1:96) [74]. The identity of components was based on their retention index relative to C_9_–C_22_ *n*-alkanes on the SPB-5 column and computer matching of spectral MS data with those from NIST MS 107 and NIST 21 libraries [75], the comparison of the fragmentation patterns with those reported in the literature [76].

### 3.3. Zebrafish and Treatment

**Figure 9:**
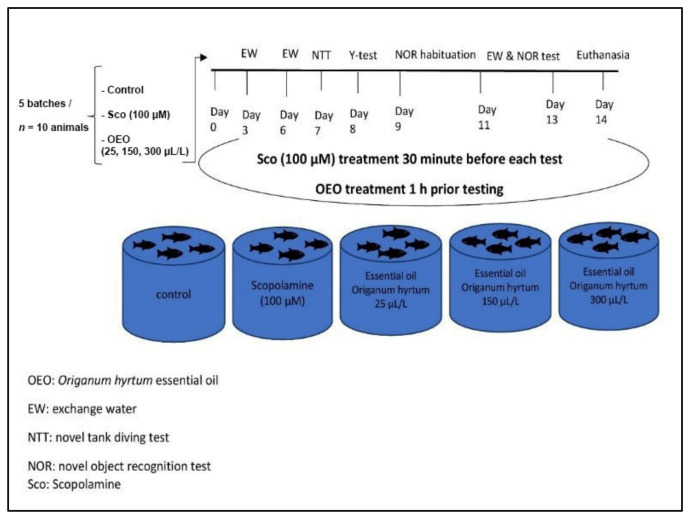
Experimental design for the *O. vulgare* ssp. *hirtum* essential oil administration in relation to scopolamine (100 μM) treatment for the study of behavioral and biochemical analysis.

Fifty adult zebrafish (*Danio rerio*) were obtained from an authorized commercial supplier (Pet Product S.R.L., Bucharest, Romania). Subjects were animals of the short-fin phenotype (3–4 months old, 3–4 cm long, 50:50 male:female ratio), which is believed to be genetically diverse and better mimic natural populations, reducing the impact of arbitrary genetic drift on inherited features [77]. Under regular conditions, zebrafish were kept in 30 L tanks filled with dechlorinated water at a maximum density of 4 fish per liter (water temperature set at 26 ± 1 °C, pH 7.0–7.2, 7.2 mg O_2_/L, conductivity 1500–1600 μS cm^−1^). Before experiments, zebrafish were acclimatized in the experimental room for at least 14 days and kept under a controlled light–dark photoperiod cycle (14/10 h, lights on 8:00 am). Animals were fed twice a day with Norwin Norvitall flake (Norwin, Gadstrup, Denmark). The animals were organized into 5 different groups (*n* = 10) designed for control, scopolamine (Sco, 100 µM), and three groups treated with *O. vulgare* ssp. *hirtum* essential oil (OEO, 25, 150, and 300 µL/L) in different tanks in a volume of 6 L each. The OEO [78] and Sco (100 µM) [2] doses were established according to previous studies. OEO (25, 150, and 300 µL/L) was administered by immersion with 1% Tween-80 solution in the zebrafish tanks, once daily, for 7 days before experiments started and throughout the 13 days of the experiment until euthanasia. Furthermore, the study was in compliance with the Ethics Committee on Animal Research of the Alexandru Ioan Cuza University of Iași, Romania, Faculty of Biology (Protocol number 02/30.06.2020), and the Directive 2010/63/EU of the European Parliament guidelines were applied to all experiments. During the experiments, no procedure caused pain or long-term injuries to the zebrafish, and no animal died during experimental testing. The experimental design is depicted in Figure 9.

### 3.4. Novel Tank-Diving Test (NTT)

The NTT methodology used in this investigation was previously reported by Cachat et al. [79] and Rosemberg et al. [80]. Animals (*n* = 50) were individually placed in a novel tank (23.9 cm along the bottom × 28.9 cm at the top × 15.1 cm high with 15.9 cm along the diagonal side, 7.4 cm wide at the top and 6.1 cm wide at the bottom) containing 1.5 L of home tank water. The behavioral activity was recorded and analyzed using ANY-Maze^®^ video tracking software (Stoelting Co., Wood Dale, IL, USA) for 6 min [78]. For further analysis, the tank was virtually divided into two areas (top and bottom) and the time spent in the top/bottom zone (s) was used to measure anxiety-related phenotype. For locomotion analysis, total distance traveled (m), the average velocity (m/s), and freezing duration (s) were calculated.

### 3.5. Y-Maze

To explore the spatial memory and the response to novelty because of OEO exposure, a Y-maze test was used, following a method previously described by Cognato et al. [81]. The Y-maze arms were designed as follows: start arm (always open); a novel arm (blocked during the first trial but opened during the second trial (test trial); and another arm (always open). Zebrafish (*n* = 50) were individually tested in a Y-maze with sides covered in black plastic self-adhesive film. Each arm included a geometric cue (square in the start arm, triangle in the novel arm, or circle in the other arm) on the side to help the fish recognize it. The Y-maze was filled with 3-L water from the home tank. For the analysis, the Y-maze center was not counted. To test the reaction to novelty, the Y-maze test consisted of two trials separated by an hour inter-trial (1 h ITI). Fish could only explore two arms (start and other) during the first trial (training, 5 min), with the third arm (novel) closed. The fish were placed back in the same starting arm with free access to all three arms for the second trial (test trial after 1 h ITI). Between groups and trials, the water from the Y-maze was changed. The behavior parameters were fully analyzed with ANY-Maze^®^ software (Stoelting Co., Wood Dale, IL, USA) by recording the time spent in each arm (% of total time), spontaneous alternation (%), and total distance traveled (m).

### 3.6. Novel Object Recognition Test (NOR)

In zebrafish, NOR is a commonly used behavioral experiment to assess memory efficiency [52]. Glass tanks (20 L, 30 × 30 x 30 cm) filled with 6 cm water from the home tank were used. There are three stages in the NOR test. During the habituation phase, each fish explored the tank without objects for 5 min twice a day (5 h between habituation sessions) over three days. On the 4^th^ day (training phase), the animals explored the tank with two similar objects (two familiar identical hard plastic red cubes objects) for 10 min. Within the test phase (1 h after the training phase), one of the familiar objects (FO, red cubes) was replaced by a novel object (NO, green cube), and the exploration time of each object was evaluated for 10 min. The exploration area was established by increasing the size of the object area once; thus, we considered exploration to be when the fish were at least 2.5 cm away from either side of the object. All data were analyzed completely with ANY-Maze software (Stoelting Co., Wood Dale, IL, USA), following the exploratory time (s) and preference percentages (time of exploration NO/time of exploration FO + time of exploration NO × 100) [3].

### 3.7. Biochemical Assays

For the biochemical assay, all zebrafish were cryoanesthetized and euthanized by decapitation [82]. The zebrafish brain samples were dissected and gently homogenized in 0.1 M potassium phosphate buffer (pH = 7.4) with 1.15% KCl using a Potter homogenizer (Heidolph Instruments, Schwabach, Germany) coupled with Cole-Parmer Servodyne Mixer (Cole-Parmer Instrument Co., Chicago, IL, USA). Homogenates were centrifuged at 906× *g* for 15 min, and the supernatant was packed in microtubes was used for the experimental assays. Protein was quantified according to the Bradford method [83]. The following parameters were quantified for oxidative stress assessment: superoxide dismutase (SOD), catalase (CAT), and glutathione peroxidase (GPX) specific activities, reduced glutathione (GSH) total content, the carbonylated proteins, and malondialdehyde (MDA) levels based of detailed methods described by Valu et al. [11]. The acetylcholinesterase (AChE) activity was quantified from the brain samples according to the previously described method by Ellman et al. [84]. All biochemical measures were performed in triplicate.

### 3.8. Statistical Analysis

The normality and homogeneity of data were checked using Shapiro–Wilk-Test. Results are expressed as means ± standard error of the mean (S.E.M). One-way analysis of variance (ANOVA) for multiple comparisons was performed to determine significant differences. When *p* < 0.05, Tukey’s post hoc multiple comparison test was employed to determine which treatment groups are different from each other. To perform statistical analyses and to represent the graphics, GraphPad Prism 8.0 (GraphPad Software, Inc., San Diego, CA, USA) was used. Correlation between behavioral results, enzymatic activities, and lipid peroxidation was estimated by the Pearson correlation coefficient (*r*).

## 4. Conclusions

The data from our study suggest that OEO administration ameliorated anxiety-like behavior and cognitive deficits measured by performance in specific behavioral tasks. OEO decreased AChE activity in the Sco-induced zebrafish model. Moreover, OEO exposure suppressed Sco-induced oxidative damage by increasing antioxidant enzymes activity and ameliorating the increased levels of protein carbonyl and MDA. The results indicate that the underlying mechanism of memory improvement involves modulations of the cholinergic system and the reduction in brain oxidative stress. Thus, these findings prove the potential of OEO as a natural, alternative treatment for anxiety and amnesia.

## Figures and Tables

**Table 1:** Chemical composition of commercial *Origanum vulgare* ssp. *hirtum* essential oil.

# ^a^	KI ^b^	KI ^c^	Class/Compound	% ^d^
**Monoterpene Hydrocarbons**				**49.68**
1	925	930	α-Thujene	1.52 (± 0.00)
2	933	939	α-Pinene	0.84 (± 0.00)
3	944	960	Thuja-2,4(10)-diene	0.02 (± 0.00)
4	949	954	Camphene	0.10 (± 0.00)
5	973	968	Verbenene	0.01 (± 0.00)
6	976	979	β-Pinene	0.14 (± 0.03)
9	988	991	β-Myrcene	2.09 (± 0.00)
10	1002	1003	α-Phellandrene	0.34 (± 0.00)
11	1009	1004	*p*-Menth-1(7),8-diene	0.11 (± 0.00)
12	1016	1017	α-Terpinene	3.51 (± 0.01)
13	1027	1025	*p*-Cymene	20.28 (± 0.03)
14	1030	1029	Limonene	0.67 (± 0.06)
16	1037	1037	*cis*-β-Ocimene	0.18 (± 0.00)
17	1048	1050	*trans*-β-Ocimene	0.09 (± 0.00)
18	1062	1060	γ-Terpinene	19.58 (± 0.08)
20	1088	1089	Terpinolene	0.20 (± 0.00)
**Oxygenated Monoterpenes**				**44.93**
15	1033	1031	1,8-Cineol	0.03 (± 0.00)
19	1069	1070	*cis*-Sabinene hydrate	0.17 (± 0.00)
22	1099	1098	*trans*-Sabinene hydrate	0.07 (± 0.00)
23	1169	1169	Borneol	0.11 (± 0.01)
24	1179	1177	Terpinen-4-ol	0.80 (± 0.00)
25	1187	1183	*p*-Cymen-8-ol	0.02 (± 0.00)
26	1196	1189	α-Terpineol	0.09 (± 0.00)
27	1203	1201	*trans*-Dihydro Carvone	0.01 (± 0.00)
28	1218	1215	*cis*-Dihydro Carvone	0.03 (± 0.00)
29	1237	1235	Thymol methyl ether	0.92 (± 0.00)
30	1248	1245	Carvacrol methyl ether	3.11 (± 0.00)
31	1260	1243	Carvone	0.03 (± 0.00)
32	1287	1289	Bornyl acetate	0.02 (± 0.00)
33	1292	1291	*p*-Cymen-7-ol	0.03 (± 0.00)
34	1306	1290	Thymol	38.82 (± 0.05)
35	1311	1299	Carvacrol	0.59 (± 0.00)
36	1358	1252	Thymol acetate	0.08 (± 0.00)
**Sesquiterpenes**				**3.94**
37	1377	1375	α-Ylangene	0.03 (± 0.00)
38	1382	1377	α-Copaene	0.07 (± 0.00)
39	1390	1388	β-Bourbonene	0.05 (± 0.00)
40	1426	1419	β-Caryophyllene	0.97 (± 0.00)
41	1435	1432	β-Copaene	0.08 (± 0.00)
42	1440	1435	β-Bergamotene	0.03 (± 0.00)
43	1460	1455	α-Humulene	0.11 (± 0.00)
44	1481	1480	γ-Muurolene	0.27 (± 0.00)
45	1485	1485	α-Amorphene	0.03 (± 0.00)
46	1492	1490	β-Selinene	0.02 (± 0.00)
47	1498	1496	γ-Amorphene	0.10 (± 0.00)
48	1504	1500	α-Muurolene	0.08 (± 0.00)
49	1512	1506	β-Bisabolene	1.27 (± 0.01)
50	1520	1514	γ-Cadinene	0.22 (± 0.00)
51	1529	1523	δ-Cadinene	0.48 (± 0.00)
52	1544	1539	α-Cadinene	0.02 (± 0.00)
53	1549	1546	α-Calacorene	0.01 (± 0.00)
54	1590	1583	Caryophyllene oxide	0.10 (± 0.00)
**Others**				**0.41**
7	978	979	1-Octen-3-ol	0.33 (± 0.03)
8	984	984	3-Octanone	0.07 (± 0.00)
21	1094	1091	Methyl benzoate	0.01 (± 0.00)
**Total**				**98.96**
**Monoterpene hydrocarbons**				**49.68**
**Oxygenated monoterpenes**				**44.93**
**Sesquiterpenes**				**3.94**
**Others**				**0.41**

^a^ The numbering refers to elution order; ^b^ Retention index (KI) relative to standard mixture of *n*-alkanes on SPB-5 column; ^c^ Literature retention index (KI); ^d^ Relative peak area percent (averages of three determinations).

## Data Availability

The data presented in this study are available on request from the corresponding author.

## References

[1] Thawkar B.S., Kaur G. (2021). Zebrafish as a promising tool for modeling neurotoxin-induced Alzheimer’s disease. Neurotox. Res..

[2] Capatina L., Todirascu-Ciornea E., Napoli E.M., Ruberto G., Hritcu L., Dumitru G. (2020). *Thymus vulgaris* essential oil protects zebrafish against cognitive dysfunction by regulating cholinergic and antioxidants systems. Antioxidants.

[3] Brinza I., Abd-Alkhalek A.M., El-Raey M.A., Boiangiu R.S., Eldahshan O.A., Hritcu L. (2020). Ameliorative effects of rhoifolin in scopolamine-induced amnesic zebrafish (*Danio rerio*) model. Antioxidants.

[4] Saleem S., Kannan R.R. (2018). Zebrafish: An emerging real-time model system to study Alzheimer’s disease and neurospecific drug discovery. Cell Death Discov..

[5] Cassar S., Adatto I., Freeman J.L., Gamse J.T., Iturria I., Lawrence C., Muriana A., Peterson R.T., Van Cruchten S., Zon L.I. (2020). Use of zebrafish in drug discovery toxicology. Chem. Res. Toxicol..

[6] Jahanshahi M., Azami N.S., Nickmahzar E. (2012). Effect of scopolamine-based amnesia on the number of astrocytes in the rat’s hippocampus. Int. J. Morphol..

[7] Pagnussat N., Almeida A.S., Marques D.M., Nunes F., Chenet G.C., Botton P.H.S., Mioranzza S., Loss C.M., Cunha R.A., Porciúncula L.O. (2015). Adenosine A 2 A receptors are necessary and sufficient to trigger memory impairment in adult mice. Br. J. Pharmacol..

[8] Oh S.-Y., Jang M.J., Choi Y.-H., Hwang H., Rhim H., Lee B., Choi C.W., Kim M.S. (2021). Central administration of afzelin extracted from *Ribes fasciculatum* improves cognitive and memory function in a mouse model of dementia. Sci. Rep..

[9] Aydin E., Hritcu L., Dogan G., Hayta S., Bagci E. (2016). The Effects of inhaled *Pimpinella peregrina* essential oil on scopolamine-induced memory impairment, anxiety, and depression in laboratory rats. Mol. Neurobiol..

[10] Mitchnick K.A., Wideman C.E., Huff A.E., Palmer D., McNaughton B.L., Winters B.D. (2018). Development of novel tasks for studying view-invariant object recognition in rodents: Sensitivity to scopolamine. Behav. Brain Res..

[11] Valu M.-V., Soare L.C., Ducu C., Moga S., Negrea D., Vamanu E., Balseanu T.-A., Carradori S., Hritcu L., Boiangiu R.S. (2021). *Hericium erinaceus* (Bull.) Pers. ethanolic extract with antioxidant properties on scopolamine-induced memory deficits in a zebrafish model of cognitive impairment. J. Fungi.

[12] Valu M.-V., Ducu C., Moga S., Negrea D., Hritcu L., Boiangiu R.S., Vamanu E., Balseanu T.A., Carradori S., Soare L.C. (2021). Effects of the Hydroethanolic Extract of *Lycopodium selago* L. on scopolamine-induced memory deficits in zebrafish. Pharmaceuticals.

[13] Sahin F., Güllüce M., Daferera D., Sökmen A., Sökmen M., Polissiou M., Agar G., Özer H. (2004). Biological activities of the essential oils and methanol extract of *Origanum vulgare* ssp. *vulgare* in the Eastern Anatolia region of Turkey. Food Control.

[14] Pezzani R., Vitalini S., Iriti M. (2017). Bioactivities of *Origanum vulgare* L.: An update. Phytochem. Rev..

[15] Salehi B., Mishra A.P., Shukla I., Sharifi-Rad M., del Contreras M.M., Segura-Carretero A., Fathi H., Nasrabadi N.N., Kobarfard F., Sharifi-Rad J. (2018). Thymol, thyme, and other plant sources: Health and potential uses. Phyther. Res..

[16] Asadbegi M., Yaghmaei P., Salehi I., Komaki A., Ebrahim-Habibi A. (2017). Investigation of thymol effect on learning and memory impairment induced by intrahippocampal injection of amyloid beta peptide in high fat diet- fed rats. Metab. Brain Dis..

[17] Leyva-López N., Gutiérrez-Grijalva E.P., Vazquez-Olivo G., Heredia J.B. (2017). Essential oils of oregano: Biological activity beyond their antimicrobial properties. Molecules.

[18] Zhang X.L., Guo Y.S., Wang C.H., Li G.Q., Xu J.J., Chung H.Y., Ye W.C., Li Y.L., Wang G.C. (2014). Phenolic compounds from *Origanum vulgare* and their antioxidant and antiviral activities. Food Chem..

[19] Ivanova D., Gerova D., Chervenkov T., Yankova T. (2005). Polyphenols and antioxidant capacity of Bulgarian medicinal plants. J. Ethnopharmacol..

[20] Avola R., Granata G., Geraci C., Napoli E., Graziano A.C.E., Cardile V. (2020). Oregano (*Origanum vulgare* L.) essential oil provides anti-inflammatory activity and facilitates wound healing in a human keratinocytes cell model. Food Chem. Toxicol..

[21] Han X., Parker T.L. (2017). Anti-inflammatory, tissue remodeling, immunomodulatory, and anticancer activities of oregano (*Origanum vulgare*) essential oil in a human skin disease model. Biochim. Open.

[22] Vujicic M., Nikolic I., Kontogianni V.G., Saksida T., Charisiadis P., Vasic B., Stosic-Grujicic S., Gerothanassis I.P., Tzakos A.G., Stojanovic I. (2016). Ethyl Acetate Extract of *Origanum vulgare* L. ssp. *hirtum* prevents streptozotocin-induced diabetes in C57BL/6 mice. J. Food Sci..

[23] Kosakowska O., Węglarz Z., Pióro-Jabrucka E., Przybył J.L., Kraśniewska K., Gniewosz M., Bączek K. (2021). Antioxidant and antibacterial activity of essential oils and hydroethanolic extracts of greek oregano (*O. vulgare* L. subsp. *hirtum* (Link) Ietswaart) and common oregano (*O. vulgare* L. subsp. *vulgare*). Molecules.

[24] Kakhki M.T., Sedaghat N., Mohsenzadeh M. (2020). Chemical composition, antioxidative, antibacterial, and time-kill activities of some selected plant essential oils against foodborne pathogenic and spoilage organisms. Vet. Res. Forum.

[25] Teixeira B., Marques A., Ramos C., Serrano C., Matos O., Neng N.R., Nogueira J.M.F., Saraiva J.A., Nunes M.L. (2013). Chemical composition and bioactivity of different oregano (*Origanum vulgare*) extracts and essential oil. J. Sci. Food Agric..

[26] Zhao Y., Yang Y.H., Wang K.B., Fan L.M., Su F.W., Ye M. (2020). Chemical composition and allelopathic potential of essential oil isolated from *Origanum vulgare*. J. Appl. Ecol..

[27] Hamada I., Al-Waili N., Aboulghazi A., Abdellaoui A., Al-Waili T., Lyoussi B. (2021). Chemical composition and antioxidant content of *Thymus vulgaris* honey and *Origanum vulgare* essential oil; their effect on carbon tetrachloride-induced toxicity. Vet. World.

[28] Amaral S.C., Pruski B.B., de Freitas S.B., Allend S.O., Ferreira M.R.A., Moreira C., Pereira D.I.B., Junior A.S.V., Hartwig D.D. (2020). *Origanum vulgare* essential oil: Antibacterial activities and synergistic effect with polymyxin B against multidrug-resistant *Acinetobacter baumannii*. Mol. Biol. Rep..

[29] Azizi Z., Ebrahimi S., Saadatfar E., Kamalinejad M., Majlessi N. (2012). Cognitive-enhancing activity of thymol and carvacrol in two rat models of dementia. Behav. Pharmacol..

[30] Sudeep H.V., Venkatakrishna K., Amritharaj, Gouthamchandra K., Reethi B., Naveen P., Lingaraju H.B., Shyamprasad K. (2021). A standardized black pepper seed extract containing β-caryophyllene improves cognitive function in scopolamine-induced amnesia model mice via regulation of brain-derived neurotrophic factor and MAPK proteins. J. Food Biochem..

[31] Napoli E., Siracusa L., Ruberto G. (2020). New Tricks for Old Guys: Recent developments in the chemistry, biochemistry, applications and exploitation of selected species from the Lamiaceae family. Chem. Biodivers..

[32] Napoli E., Giovino A., Carrubba A., How Yuen Siong V., Rinoldo C., Nina O., Ruberto G. (2020). Variations of essential oil constituents in oregano (*Origanum vulgare* subsp. *viridulum* (= *O. heracleoticum*) over cultivation cycles. Plants.

[33] Licata M., Tuttolomondo T., Dugo G., Ruberto G., Leto C., Napoli E.M., Rando R., Fede M.R., Virga G., Leone R. (2015). Study of quantitative and qualitative variations in essential oils of Sicilian oregano biotypes. J. Essent. Oil Res..

[34] Mancini E., Camele I., Elshafie H.S., De Martino L., Pellegrino C., Grulova D., De Feo V. (2014). Chemical composition and biological activity of the essential oil of *Origanum vulgare* ssp. *hirtum* from different areas in the southern Apennines (Italy). Chem. Biodivers..

[35] Stešević D., Jaćimović Ž., Šatović Z., Šapčanin A., Jančan G., Kosović M., Damjanović-Vratnica B. (2018). Chemical characterization of wild growing *Origanum vulgare* populations in Montenegro. Nat. Prod. Commun..

[36] Pasias I.N., Ntakoulas D.D., Raptopoulou K., Gardeli C., Proestos C. (2021). Chemical composition of essential oils of aromatic and medicinal herbs cultivated in Greece—Benefits and drawbacks. Foods.

[37] Qiao Y., Yu Z., Bai L., Li H., Zhang S., Liu J., Gao Z., Yang X. (2021). Chemical composition of essential oils from *Thymus mongolicus*, *Cinnamomum verum*, and *Origanum vulgare* and their acaricidal effects on *Haemaphysalis longicornis* (Acari: Ixodidae). Ecotoxicol. Environ. Saf..

[38] Zhao Y., Yang Y.H., Ye M., Wang K.B., Fan L.M., Su F.W. (2021). Chemical composition and antifungal activity of essential oil from *Origanum vulgare* against *Botrytis cinerea*. Food Chem..

[39] Wong K., Elegante M., Bartels B., Elkhayat S., Tien D., Roy S., Goodspeed J., Suciu C., Tan J., Grimes C. (2010). Analyzing habituation responses to novelty in zebrafish (*Danio rerio*). Behav. Brain Res..

[40] Blaser R.E., Rosemberg D.B. (2012). Measures of anxiety in zebrafish (*Danio rerio*): Dissociation of black/white preference and novel tank test. PLoS ONE.

[41] Kalueff A.V., Cachat J.M. (2011). Zebrafish Models in Neurobehavioral Research.

[42] Amiresmaeili A., Roohollahi S., Mostafavi A., Askari N. (2018). Effects of oregano essential oil on brain TLR4 and TLR2 gene expression and depressive-like behavior in a rat model. Res. Pharm. Sci..

[43] Abbasi-Maleki S., Kadkhoda Z., Taghizad-Farid R. (2020). The antidepressant-like effects of *Origanum majorana* essential oil on mice through monoaminergic modulation using the forced swimming test. J. Tradit. Complement. Med..

[44] Rezaie A., Mousavi G., Nazeri M., Jafari B., Ebadi A., Ahmadeh C., Habibi E. (2011). Comparative study of sedative, pre-anesthetic and anti-anxiety effect of *Origanum majorana* extract with diazepam on rats. Res. J. Biol. Sci..

[45] Mombeini T., Mazloumi S., Shams J. (2015). Pharmacological effects of *Origanum vulgare* L. in the elevated plus-maze and open field tests in the rat. J. Basic Clin. Pathophysiol..

[46] Da Machado K.C., Paz M.F.C.J., de Oliveira Santos J.V., da Silva F.C.C., Tchekalarova J.D., Salehi B., Islam M.T., Setzer W.N., Sharifi-Rad J., de Castro e Sousa J.M. (2020). Anxiety therapeutic interventions of β-caryophyllene: A laboratory-based study. Nat. Prod. Commun..

[47] Hanrahan J.R., Chebib M., Johnston G.A.R. (2011). Flavonoid modulation of GABA A receptors. Br. J. Pharmacol..

[48] Bianchini A.E., Garlet Q.I., Da Cunha J.A., Bandeira Junior G., Brusque I.C.M., Salbego J., Heinzmann B.M., Baldisserotto B. (2017). Monoterpenoids (thymol, carvacrol and S-(+)-linalool) with anesthetic activity in silver catfish (*Rhamdia quelen*): Evaluation of acetylcholinesterase and GABAergic activity. Brazilian J. Med. Biol. Res..

[49] Dougnon G., Ito M. (2021). Role of Ascaridole and p-cymene in the sleep-promoting effects of *Dysphania ambrosioides* essential oil via the GABAergic system in a ddY mouse inhalation model. J. Nat. Prod..

[50] Wang Z.J., Heinbockel T. (2018). Essential oils and their constituents targeting the GABAergic system and sodium channels as treatment of neurological diseases. Molecules.

[51] Brinza I., Boiangiu R.S., Hancianu M., Cioanca O., Orhan I.E., Hritcu L. (2021). Bay leaf (*Laurus nobilis* L.) incense improved scopolamine-induced amnesic rats by restoring cholinergic dysfunction and brain antioxidant status. Antioxidants.

[52] Gaspary K.V., Reolon G.K., Gusso D., Bonan C.D. (2018). Novel object recognition and object location tasks in zebrafish: Influence of habituation and NMDA receptor antagonism. Neurobiol. Learn. Mem..

[53] Ghaderi A., Karimi S.A., Talaei F., Shahidi S., Faraji N., Komaki A. (2020). The effects of aqueous extract of *Origanum vulgare* on learning and memory in male rats. J. Herbmed Pharmacol..

[54] Haghpanah T., Bezanjani E.K., Khaki M.R.A., Sheibani V., Abbasnejad M., Ardakani Y.M. (2011). Effect of intra-hippocampal injection of *Origanum vulgare* L. ssp. *viridis* leaf extract on spatial learning and memory consolidation. KAUMS J. (FEYZ).

[55] Maryam A., Farhad V., Bagher S. (2021). Investigation of Origanum vulgare L. Leaf Extract on Ethanol-Induced Impairment of Working Memory on Male Rat.

[56] Sheibani V., Afarinesh M., Hajializadeh Z., Abbasnejad M., Haghpanah T., Arabnezhad R., Sepehri G. (2011). Evaluation of *Origanum vulgare* L. ssp. *viridis* leaves extract effect on discrimination learning and LTP induction in the CA1 region of the rat hippocampus. Iran. J. Basic Med. Sci..

[57] Seifi-Nahavandi B., Yaghmaei P., Ahmadian S., Ghobeh M., Ebrahim-Habibi A. (2020). Cymene consumption and physical activity effect in Alzheimer’s disease model: An in vivo and in vitro study. J. Diabetes Metab. Disord..

[58] Kim K., Bu Y., Jeong S., Lim J., Kwon Y., Cha D.S., Kim J., Jeon S., Eun J., Jeon H. (2006). Memory-enhancing effect of a supercritical carbon dioxide fluid extract of the needles of *Abies koreana* on scopolamine-induced amnesia in mice. Biosci. Biotechnol. Biochem..

[59] Carrasco A., Perez E., Cutillas A.-B., Martinez-Gutierrez R., Tomas V., Tudela J. (2016). *Origanum vulgare* and *Thymbra capitata* essential oils from Spain: Determination of aromatic profile and bioactivities. Nat. Prod. Commun..

[60] Lombrea A., Antal D., Ardelean F., Avram S., Pavel I.Z., Vlaia L., Mut A.-M., Diaconeasa Z., Dehelean C.A., Soica C. (2020). A Recent insight regarding the phytochemistry and bioactivity of *Origanum vulgare* L. essential oil. Int. J. Mol. Sci..

[61] Sarikurkcu C., Zengin G., Oskay M., Uysal S., Ceylan R., Aktumsek A. (2015). Composition, antioxidant, antimicrobial and enzyme inhibition activities of two *Origanum vulgare* subspecies (subsp. *vulgare* and subsp. *hirtum*) essential oils. Ind. Crops Prod..

[62] Sohn E., Kim Y.J., Kim J.-H., Jeong S.-J. (2021). *Ficus erecta* Thunb leaves alleviate memory loss induced by scopolamine in mice via regulation of oxidative stress and cholinergic system. Mol. Neurobiol..

[63] Paloczi J., Varga Z.V., Hasko G., Pacher P. (2018). Neuroprotection in oxidative stress-related neurodegenerative diseases: Role of endocannabinoid system modulation. Antioxid. Redox Signal..

[64] Wang X.-C., Xu Y.-M., Li H.-Y., Wu C.-Y., Xu T.-T., Luo N.-C., Zhang S.-J., Wang Q., Quan S.-J. (2018). Jiao-Tai-Wan improves cognitive dysfunctions through cholinergic pathway in scopolamine-treated mice. Biomed. Res. Int..

[65] Sharifi-Rigi A., Heidarian E., Amini S.A. (2019). Protective and anti-inflammatory effects of hydroalcoholic leaf extract of *Origanum vulgare* on oxidative stress, TNF-α gene expression and liver histological changes in paraquat-induced hepatotoxicity in rats. Arch. Physiol. Biochem..

[66] Zou Y., Wang J., Peng J., Wei H. (2016). Oregano Essential Oil Induces SOD1 and GSH Expression through Nrf2 activation and alleviates hydrogen peroxide-induced oxidative damage in IPEC-J2 Cells. Oxid. Med. Cell. Longev..

[67] Sun Q.-F., Chen S.-X., Tang Z.-F., Song X.-Y., Jing F., Wu H.-T., Ding Z.-Y., El-kott A., Massoud D., Khalifa H. (2021). *Origanum vulgare* L. leaf extract alleviates finasteride-induced oxidative stress in mouse liver and kidney. Asian Pac. J. Trop. Biomed..

[68] Namiecińska E., Sadowska B., Więckowska-Szakiel M., Dołęga A., Pasternak B., Grazul M., Budzisz E. (2019). Anticancer and antimicrobial properties of novel η6-p-cymene ruthenium(II) complexes containing a N,S-type ligand, their structural and theoretical characterization. RSC Adv..

[69] Siddiqui M.N., Redhwi H.H., Tsagkalias I., Vouvoudi E.C., Achilias D.S. (2021). Development of bio-composites with enhanced antioxidant activity based on poly(lactic acid) with thymol, carvacrol, limonene, or cinnamaldehyde for active food packaging. Polymers.

[70] Formiga R.d.O., Júnior E.B.A., Vasconcelos R.C., Guerra G.C.B., de Araújo A.A., de Carvalho T.G., Garcia V.B., Junior R.F.d.A., Gadelha F.A.A.F., Vieira G.C. (2020). P-cymene and rosmarinic acid ameliorate tnbs-induced intestinal inflammation upkeeping zo-1 and muc-2: Role of antioxidant system and immunomodulation. Int. J. Mol. Sci..

[71] Memari-Tabrizi E.F., Yousefpour-Dokhanieh A., Babashpour-Asl M. (2021). Foliar-applied silicon nanoparticles mitigate cadmium stress through physio-chemical changes to improve growth, antioxidant capacity, and essential oil profile of summer savory (Satureja hortensis L.). Plant Physiol. Biochem..

[72] Morshedloo M.R., Mumivand H., Craker L.E., Maggi F. (2018). Chemical composition and antioxidant activity of essential oils in *Origanum vulgare* subsp. *gracile* at different phenological stages and plant parts. J. Food Process. Preserv..

[73] Qneibi M., Jaradat N., Hawash M., Zaid A.N., Natsheh A.R., Yousef R., AbuHasan Q. (2019). The neuroprotective role of *Origanum syriacum* L. and *Lavandula dentata* L. essential oils through their effects on AMPA receptors. Biomed. Res. Int..

[74] Napoli E.M., Curcuruto G., Ruberto G. (2009). Screening the essential oil composition of wild Sicilian oregano. Biochem. Syst. Ecol..

[75] NIST Standard Reference Database 1A. https://www.nist.gov/system/files/documents/srd/NIST1aVer22Man.pdf.

[76] Sparkman O.D. (2005). Identification of essential oil components by gas chromatography/quadrupole mass spectroscopy Robert P. Adams. J. Am. Soc. Mass Spectrom..

[77] Franscescon F., Souza T.P., Müller T.E., Michelotti P., Canzian J., Stefanello F.V., Rosemberg D.B. (2021). Taurine prevents MK-801-induced shoal dispersion and altered cortisol responses in zebrafish. Prog. Neuro-Psychopharmacol. Biol. Psychiatry.

[78] Dos Santos A.C., Junior G.B., Zago D.C., Zeppenfeld C.C., da Silva D.T., Heinzmann B.M., Baldisserotto B., da Cunha M.A. (2017). Anesthesia and anesthetic action mechanism of essential oils of *Aloysia triphylla* and *Cymbopogon flexuosus* in silver catfish (*Rhamdia quelen*). Vet. Anaesth. Analg..

[79] Cachat J.M., Canavello P.R., Elkhayat S.I., Bartels B.K., Hart P.C., Elegante M.F., Beeson E.C., Laffoon A.L., Haymore W.A.M., Tien D.H. (2011). Video-aided analysis of zebrafish locomotion and anxiety-related behavioral responses. Neuromethods.

[80] Rosemberg D.B., Rico E.P., Mussulini B.H.M., Piato Â.L., Calcagnotto M.E., Bonan C.D., Dias R.D., Blaser R.E., Souza D.O., de Oliveira D.L. (2011). Differences in spatio-temporal behavior of zebrafish in the open tank paradigm after a short-period confinement into dark and bright environments. PLoS ONE.

[81] De Cognato G.P., Bortolotto J.W., Blazina A.R., Christoff R.R., Lara D.R., Vianna M.R., Bonan C.D. (2012). Y-Maze memory task in zebrafish (*Danio rerio*): The role of glutamatergic and cholinergic systems on the acquisition and consolidation periods. Neurobiol. Learn. Mem..

[82] Brinza I., Ayoub I.M., Eldahshan O.A., Hritcu L. (2021). Baicalein 5,6-dimethyl ether prevents memory deficits in the scopolamine zebrafish model by regulating cholinergic and antioxidant systems. Plants.

[83] Kielkopf C.L., Bauer W., Urbatsch I.L. (2020). Bradford assay for determining protein concentration. Cold Spring Harb. Protoc..

[84] Ellman G.L., Courtney K.D., Andres V.J., Featherstone R.M., Feather-Stone R. (1961). A new and rapid colorimetric determination of acetylcholinesterase activity. Biochem. Pharmacol..

